# Supplementary material for: Perinatal deaths attributable to congenital heart defects in Hunan Province, China, 2016–2020
Source: PLoS One. 2024 Jun 13;19(6):e0304615. doi: 10.1371/journal.pone.0304615 (PMC11175501; doi:10.1371/journal.pone.0304615)
Supplement: Attachment — Submitted filename: Response to Reviewers.docx [file pone.0304615.s001.docx]

**PONE-D-23-39650**

**Perinatal deaths from congenital heart defects in Hunan Province, China, 2016-2020**

PLOS ONE

**There are two major issues which need to be addressed:**

- It is unclear which conclusions can be drawn from this analysis, as the perinatal mortality in CHD children seems mainly driven by selective TOPs. It might therefore be more appropriate to re-do the whole analysis with selective TOP instead of perinatal mortality as the main outcome, and to discuss the strikingly high rates of selective TOP in CHD pregnancies in Hunan (or China). Results for perinatal mortality might be shown within a sensitivity analysis.

**Response**: Thanks for your suggestion very much.

According to your suggestion, we have done some revisions, but we did not re-do the whole analysis with selective TOP instead of perinatal mortality as the main outcome. The following are our main reasons:

1. Although the perinatal mortality in CHD children seems mainly driven by selective TOPs, the main cause of perinatal deaths was CHDs or other severe birth defects, not selective TOP. These perinatal deaths would not have been preventable without the selective TOP. And the primary reason doctors adopt selective TOP is to alleviate the suffering of the pregnant woman and their families.

2. Selective TOP is also a sensitive topic in China, so we have tried our best to avoid statements about it in the manuscript.

If further revisions are required, we will do so as soon as possible.

- Much more information is needed to understand the design of the Birth Defects Surveillance System, e.g. how, when and where where the participating women exactly recruited, what was the participation rate, did the participating and non-participating women differ with respect to demographic characteristics and birth outcomes, how were they followed up with respect to their birth outcomes, was there loss of follow-up, which information was collected and how etc...

**Response**: Thanks for your suggestion very much.

According to your suggestion, we have revised the manuscript in **lines 92-151 (Methods)**

When submitting your revision, we need you to address these additional requirements.

1. Please ensure that your manuscript meets PLOS ONE's style requirements, including those for file naming. The PLOS ONE style templates can be found at

https://journals.plos.org/plosone/s/file?id=wjVg/PLOSOne_formatting_sample_main_body.pdf and

https://journals.plos.org/plosone/s/file?id=ba62/PLOSOne_formatting_sample_title_authors_affiliations.pdf

2. We noticed you have some minor occurrence of overlapping text with the following previous publication(s), which needs to be addressed:

https://bmcpregnancychildbirth.biomedcentral.com/articles/10.1186/s12884-023-06092-5

https://www.nature.com/articles/s41598-023-47741-1

In your revision ensure you cite all your sources (including your own works), and quote or rephrase any duplicated text outside the methods section. Further consideration is dependent on these concerns being addressed.

**Response**: Thanks for your suggestion very much.

According to your suggestion, we have revised the manuscript in **lines 52-91 (Introduction), 152-224 (Results), and 231-320 (Discussion).**

3. We note that you have indicated that there are restrictions to data sharing for this study. For studies involving human research participant data or other sensitive data, we encourage authors to share de-identified or anonymized data. However, when data cannot be publicly shared for ethical reasons, we allow authors to make their data sets available upon request. For information on unacceptable data access restrictions, please see http://journals.plos.org/plosone/s/data-availability#loc-unacceptable-data-access-restrictions.

Before we proceed with your manuscript, please address the following prompts:

a) If there are ethical or legal restrictions on sharing a de-identified data set, please explain them in detail (e.g., data contain potentially identifying or sensitive patient information, data are owned by a third-party organization, etc.) and who has imposed them (e.g., a Research Ethics Committee or Institutional Review Board, etc.). Please also provide contact information for a data access committee, ethics committee, or other institutional body to which data requests may be sent.

b) If there are no restrictions, please upload the minimal anonymized data set necessary to replicate your study findings to a stable, public repository and provide us with the relevant URLs, DOIs, or accession numbers. Please see http://www.bmj.com/content/340/bmj.c181.long for guidelines on how to de-identify and prepare clinical data for publication. For a list of recommended repositories, please see https://journals.plos.org/plosone/s/recommended-repositories. You also have the option of uploading the data as Supporting Information files, but we would recommend depositing data directly to a data repository if possible.

Please update your Data Availability statement in the submission form accordingly.

**Response**: Thanks for your suggestion very much.

According to your suggestion, we have revised the manuscript in **lines 119-123 (Ethics approval and consent to participate) and 336-338 (Data Availability).**

**Additional Editor Comments:**

**Whole manuscript:**

- Please replace "multivariate" by "multivariable".

**Response**: Thanks for your suggestion very much.

According to your suggestion, we have revised the manuscript in **lines 26, 33, 82, 86, 143, 203, 223, 226.**

- Speaking of "risk factors" and "protective factors" is somewhat misleading, as the authors show associations, but not causal relations.

**Response**: Thanks for your suggestion very much.

According to your suggestion, we have revised the manuscript in **lines 27-28, 75-76, and 143-147.**

- The interpretation of the ORs is not straightforward and should be explained shortly both in the Abstract and in the main text.

**Response**: Thanks for your suggestion very much.

According to your suggestion, we have revised the manuscript in **lines 20-28 (Abstract - Methods) and 139-151 (Abstract - Statistical analysis).**

- The first and last sentence of the Conclusions (both in the abstract and twice in the main text) are superfluous and should therefore be omitted.

**Response**: Thanks for your suggestion very much.

According to your suggestion, we have revised the manuscript in **lines 321-327 (Conclusion).**

- Suggest to replace per thousand by percent throughout the manuscript (including table 1) to make the proportion numbers directly comparable to each other.

**Response**: Thanks for your suggestion very much.

According to your suggestion, we have revised the manuscript in **lines 166-167 (Table 1) and 182-185 (Table 2).**

- In the spirit of Open and Reproducible Science, the analysis code should be made available in an online repository together with a data dictionary, and the respective URL should be mentioned in the Methods section.

**Response**: Thanks for your suggestion very much.

According to your suggestion, we have revised the manuscript in **lines 139-151 (Statistical analysis) and 336-338 (Data Availability).**

**Abstract:**

- The sentence "Multivariate logistic regression analysis showed..." is awkward and should be revised, e.g. "In stepwise logistic regression analysis, the following variables were selected for the final model: ..."

**Response**: Thanks for your suggestion very much.

According to your suggestion, we have revised the manuscript in **lines 33-35 (Abstract) and 213-215 (Results).**

- ORs and 95% CIs should be reported for all predictors; p-values should be rather left out.

**Response**: Thanks for your suggestion very much.

According to your suggestion, we have revised the manuscript in **lines 29-44 (Abstract - Results) and 186-230 (Results).**

- It is difficult to understand the meaning of "diagnosed in the postnatal period" and "low gestational age of diagnosis". This should be described in a clearer way.

**Response**: Thanks for your suggestion very much.

According to your suggestion, we have revised the manuscript in **lines 234 and 293.**

**Main text:**

- l. 60: Suggest to replace "were" by "are".

**Response**: Thanks for your suggestion very much.

According to your suggestion, we have revised the manuscript in **lines 53-54.**

- l. 63: What is meant by "accepted prevalence"?

**Response**: Thanks for your suggestion very much.

According to your suggestion, we have revised the manuscript in **lines 55-56.**

- l. 70: Abbreviation "WHO" should be explained at first appearance.

**Response**: Thanks for your suggestion very much.

According to your suggestion, we have revised the manuscript in **lines 62-64.**

- l. 74-93: Instead of just counting up these studies, they should be set in context to each other and to the research question. The same applies for the limitations.

**Response**: Thanks for your suggestion very much.

According to your suggestion, we have revised the manuscript in **lines 66-87.**

- l. 106-109: What was the rationale to choose exactly these 8 variables? Ideally, some references should be added here. Additionally, this sentence should rather be put into the "statistical analysis" section.

**Response**: Thanks for your suggestion very much.

According to your suggestion, we have revised the manuscript in **lines 147-150 (Methods -** **Statistical analysis) and 206-213 (Results).**

- Maternal BMI, gestational weight gain as well as gestational and pre-gestational diabetes should be added as potential predictors, if available. If not, the lack of these variables should be discussed as a potential limitation.

**Response**: Thanks for your suggestion very much.

According to your suggestion, we have revised the manuscript in **lines 310-320 (Discussion - potential limitation).**

- l. 120-122: How (and why) were the anonymized data deidentified?

**Response**: Thanks for your suggestion very much.

According to your suggestion, we have revised the manuscript in **lines 118-132 (Ethics approval and consent to participate).**

- l. 122: Which experiments are meant here?

**Response**: Thanks for your suggestion very much.

According to your suggestion, we have revised the manuscript in **lines 118-132 (Ethics approval and consent to participate).**

- Please add a table showing demographic characteristics (including gestational age and numbers of preterm births) for the whole dataset. Were there any missing data in these variables, or if not, why not?

**Response**: Thanks for your suggestion very much.

According to your suggestion, we have revised the manuscript in **lines 202-204 (Table 3. Results of the univariate analysis).**

Some demographic characteristics were not in the table due to data limitation, such as variables were not included in the case cards or missing data.

- Table 1: It would be helpful to the reader to explain the difference of "PMR of" and "PMR attributable to" (or TOP instead of PMR in the revised version) in the table legend.

**Response**: Thanks for your suggestion very much.

According to your suggestion, we have revised the manuscript in **lines 71, 72, 81, 153, 166, 168, 181, 182, 187, 193, 195, 198, 239-243, 252, 253, 264, 268, 279, and 325.**

In the revised manuscript, we revised all "PMR attributable to" to "PMR of".

- Table 4: This information should be given in the main text instead of adding a table for this.

**Response**: Thanks for your suggestion very much.

As it is rather cumbersome to express it in words, we propose to keep the table.

If further revisions are required, we will do so as soon as possible.

- l. 289-294: This paragraph is weak and should be replaced by a thorough discussion about limitations and strengths of this analysis.

**Response**: Thanks for your suggestion very much.

According to your suggestion, we have revised the manuscript in **lines 310-320 (limitations) and 232-238 (strengths).**

And in the Introduction and Discussion sections, we also presented the strengths of this study.

- Additionally, another paragraph might be added which discusses a) potential ways to further reduce PMR / TOP in CHD pregnancies in China and b) the generalizability of these results to whole China and to other countries.

**Response**: Thanks for your suggestion very much.

According to your suggestion, we have revised the manuscript in **lines 324-327.**

**Reviewers' comments:**

**Reviewer's Responses to Questions**

**Comments to the Author**

**5. Review Comments to the Author**

**Reviewer #1:** The manuscript described the perinatal mortality rates (PMR) of congenital heart defects (CHDs) and identified risk factors for perinatal deaths attributable to CHDs. The prevalence of CHDs was 4.91‰ (95%CI: 4.76-5.06).The total PMR was 0.88% (95%CI: 0.86-0.90), and the PMR of CHDs was 23.46%. This manuscript provides an appropriate study design and performance.

**Major points**

1.I suggest the author to provide more specific statistical methods and results，e.g. the inclusion criteria of variables in logistic regression analysis, VIF between variables? p values in tables?

**Response**: Thanks for your suggestion very much.

According to your suggestion, we have revised the manuscript in **lines 139-151 (Statistical analysis) and 152-230 (Results).**

2. Cases of BD included CHD?

**Response**: Thanks for your suggestion very much.

According to your suggestion, we have revised the manuscript in **lines 153-165.**

In this study, cases of BD included CHD.

3. Is there difference between years?

**Response**: Thanks for your suggestion very much.

According to your suggestion, we have revised the manuscript in **lines 163-165 (Results).**

In this study, there difference in PMRs of CHDs between years.

**Reviewer #2:** This is a work on the perinatal deaths from congenital heart defects in Hunan Province, China. The authors describe the perinatal mortality rates of congenital heart defects and use multivariate logistic regression to identify possible risk factors.

The manuscript and the results are well organised. Please find my comments below:

- Lines 41-49: Mulitvariate analysis results should be more consistent. Please use ORs, 95%Cis and p values (in their exact values) to describe the multivariate model estimates.

**Response**: Thanks for your suggestion very much.

According to your suggestion, we have revised the manuscript in **lines 29-44 (Abstract - Results).**

- Lines 74-93: The first name of the author is used when mentioned the references 16, 17, 14. Please revise.

**Response**: Thanks for your suggestion very much.

According to your suggestion, we have revised the manuscript in **lines 69-78.**

- Line 146: The reference for SPSS version 18.0 is wrong. For versions 18 and earlier "SPSS Inc. in Chicago" should be used instead of "IBM Corp., NY, USA". (can be found in: https://www.ibm.com/support/pages/how-cite-ibm-spss-statistics-or-earlier-versions-spss).

**Response**: Thanks for your suggestion very much.

According to your suggestion, we have revised the manuscript in **lines 151.**

- Lines 189-190 (and throught the text): Please use the exact value of p value (only if p=0.000 then it should be given as p<0.001).

**Response**: Thanks for your suggestion very much.

According to your suggestion, we have revised the manuscript in **lines 152-230 (Results).**

- Lines 197-206 Same as the first comment, please use ORs, 95%Cis and p values (in their exact values) to describe the multivariate model estimates.

**Response**: Thanks for your suggestion very much.

According to your suggestion, we have revised the manuscript in **lines 206-234.**

- Table 5: P values should be given in their exact values with three decimals. Again, if a p values=0.00 then it should be given as p<0.001.

**Response**: Thanks for your suggestion very much.

According to your suggestion, we have revised the manuscript in **lines 228-230 (Table 5).**
