# Supplementary material for: Perinatal deaths attributable to congenital heart defects in Hunan Province, China, 2016–2020
Source: PLoS One. 2024 Jun 13;19(6):e0304615. doi: 10.1371/journal.pone.0304615 (PMC11175501; doi:10.1371/journal.pone.0304615)
Supplement: Attachment — Submitted filename: Response to Reviewers.docx [file pone.0304615.s002.docx]

**PONE-D-23-39650R1**

**Perinatal deaths attributable to congenital heart defects in Hunan Province, China, 2016-2020**

**Additional Editor Comments:**

The authors have done well in revising and improving their manuscript. However, a few issues remain to be addressed, please see below:

- Abstract: Suggest to write "In stepwise logistic regression analysis, perinatal deaths...", i.e. omit the statement which variables were selected.

**Response**: Thanks for your suggestion very much.

According to your suggestion, we revised it in **lines 37-38.**

- l. 38-44: Rather than giving the ORs of all categories it might be more relevant to the reader to mention the associations of PMR with other variables such as time of diagnosis.

**Response**: Thanks for your suggestion very much.

According to your suggestion, we revised it in **lines 38-42.**

- Unfortunately, the paragraph in l. 66-87 is still weak both with respect to English wording and with respect to content. It is also unclear what is meant by "unrepresentative hospitals" or "some studies need to be updated". The text might improve if the authors will remove the counting ("first" to "fifth") and integrate the references mentioned before directly into their arguments.

**Response**: Thanks for your suggestion very much.

According to your suggestion, we revised it in **lines 63-83.**

- In general, proof-reading of the whole manuscript by an English native speaker might be advisable.

**Response**: Thanks for your suggestion very much.

Due to time and financial constraints, we asked people who have lived and studied in the United States to revise the entire text.

We hope that these revisions meet the requirements.

If further revisions are required, we will do it as soon as possible.

- l. 96: What were the criterions to define "representative hospitals"? Out of how many hospitals were these 52 hospitals chosen?

**Response**: Thanks for your suggestion very much.

According to your suggestion, we revised it in **lines 90-98.**

- l. 189-192: Suggest to write "Compared to senior high school as maternal education level..."

**Response**: Thanks for your suggestion very much.

According to your suggestion, we revised it in **lines 179-182.**

- l. 38-40 and 193: add "years"

**Response**: Thanks for your suggestion very much.

According to your suggestion, we revised it in **lines 39-40 and 198-201.**

- l. 193-194: These PMRs are not attributed to the age categories in the same way as in table 3.

**Response**: Thanks for your suggestion very much.

According to your suggestion, we revised it in **lines 183-185.**

- In general, the text should repeat only a small selection of the numbers given in the tables. It is enough to mention the associations in a qualitative way (e.g. "maternal age was negatively associated with PMR...") and refer to the respective tables. All numbers which remain in the text should be thoroughly checked for consistence with the tables.

**Response**: Thanks for your suggestion very much.

According to your suggestion, we revised it in **lines 183-185.**

- l. 196: The weeks should be put out of the brackets, as the sentence makes no sense without this information.

**Response**: Thanks for your suggestion very much.

According to your suggestion, we revised it in **lines 183-185.**

- Table 4 is superfluous.

**Response**: Thanks for your suggestion very much.

According to your suggestion, we deleted table 4.

- l. 216-224: It would be sufficient to mention that the results from table 5 were quite similar to those from table 3, i.e. mutual adjustment did not affect the associations considerably, apart from the obervsation that the OR for rural area was somewhat attenuated. The latter might be mentioned in the discussion with the interpretation that this analysis apparently covered some, but not all aspects which account for higher PMRs in rural areas.

**Response**: Thanks for your suggestion very much.

According to your suggestion, we revised it in **lines 250-254.**

- l. 235: Suggest to add "To our knowledge,..."

**Response**: Thanks for your suggestion very much.

According to your suggestion, we revised it in **lines 212-213.**

- l. 239-241: This result needs to be set in context. Is Hunan a less developed area in China?

**Response**: Thanks for your suggestion very much.

According to your suggestion, we revised it in **lines 218-219 and 224-226.**

- l. 248-250: Is there any evidence to support this statement, e.g. other medical improvements during this period in Hunan?

**Response**: Thanks for your suggestion very much.

According to your suggestion, we revised it in **lines 227-229.**

- l. 265-266 and 293-295: I don't understand these sentences, please explain in more detail.

**Response**: Thanks for your suggestion very much.

According to your suggestion, we revised it in **lines 237-254 and 271-278.**

- l. 279-283: This argument seems to contradict your argument from the Response Letter that these perinatal deaths would not have been preventable without the selective TOP. Please explain.

**Response**: Thanks for your suggestion very much.

According to your suggestion, we revised it in **lines 255-261.**

- l. 64-65, 207-213, 239-241, 257-263, 298, 324-327: These sentences seem superfluous.

**Response**: Thanks for your suggestion very much.

According to your suggestion, we deleted it.

- The Methods section still lacks an URL were the analysis code can be found.

**Response**: Thanks for your suggestion very much.

[Multivariate logistic regression analysis of risk factors for birth defects: a study from population-based surveillance data - PubMed (nih.gov)](https://pubmed.ncbi.nlm.nih.gov/38622560/)

[Epidemiology of congenital polydactyly and syndactyly in Hunan Province, China - PubMed (nih.gov)](https://pubmed.ncbi.nlm.nih.gov/38521899/)

We have published several similar articles and all the information needed has been provided.

Please give us more detailed tips.
