# Supplementary material for: Perinatal deaths attributable to congenital heart defects in Hunan Province, China, 2016–2020
Source: PLoS One. 2024 Jun 13;19(6):e0304615. doi: 10.1371/journal.pone.0304615 (PMC11175501; doi:10.1371/journal.pone.0304615)
Supplement: Attachment — Submitted filename: Response to Reviewers.docx [file pone.0304615.s003.docx]

**PONE-D-23-39650R2**

**Perinatal deaths attributable to congenital heart defects in Hunan Province, China, 2016-2020**

**Additional Editor Comments:**

- l. 23: The sentence should read "...whose mothers delivered...".

**Response**: Thanks very much for your suggestion.

According to your suggestion, we revised it in **lines 23-24.**

- l. 24-30: Suggest to condense these sentences as follows: "Surveillance data included demographic characteristics such as sex, residence, maternal age, and other key information, and were used to calculate prevalence of CHDs and perinatal mortality rates (PMR) with 95% confidence intervals (CI)."

**Response**: Thanks very much for your suggestion.

According to your suggestion, we revised it in **lines 24-26.**

- l. 40-41: "OR>1" is too imprecise and should be replaced by the calculated ORs with 95% CIs.

**Response**: Thanks very much for your suggestion.

According to your suggestion, we revised it in **lines 35-44.**

- l. 44 and 301: Suggest to write "Perinatal deaths were common in CHDs in Hunan in 2016-2020."

**Response**: Thanks very much for your suggestion.

According to your suggestion, we revised it in **lines 46 and 312.**

- l. 68: It should be made clear that reference 9 was written by the same authors based on the same dataset, and it should be explained in which aspects this new paper overlaps with the old one and what it adds.

**Response**: Thanks very much for your suggestion.

According to your suggestion, we revised it in **lines 71-76.**

- l. 83 (and other places): "Multivariate" should be replaced by "multivariable".

**Response**: Thanks very much for your suggestion.

According to your suggestion, we revised it in **lines 91, 92, 142, 148 and 223.**

- l. 121-122: I still don't understand why and how patient records were "deidentified" given that they were anonymized anyway. Can this sentence be omitted? Otherwise pleas add some details.

**Response**: Thanks very much for your suggestion.

According to your suggestion, we revised it in **lines 128.**

- l. 131: Suggest to write "Prevalence and PMR of specific CHDs...".

**Response**: Thanks very much for your suggestion.

According to your suggestion, we revised it in **lines 137.**

- l. 149: Which Government? Suggest to write: "The Hunan Provincial Health Commission routinely collected surveillance data based on the Hunan Maternal and Child Health Surveillance Manual developed by...".

**Response**: Thanks very much for your suggestion.

According to your suggestion, we revised it in **lines 124-125.**

- l. 190-195: Suggest to omit the first two sentences and just write: "Starting with all variables from table 3, the following variables were selected for the final model in stepwise logistic regression analysis: ..."

**Response**: Thanks very much for your suggestion.

According to your suggestion, we revised it in **lines 197-199.**

- l. 196: In order to make the difference to the previous analysis clearer, the sentence might rather begin with "In the logistic regression model with mutual adjustment, perinatal deaths..."

**Response**: Thanks very much for your suggestion.

According to your suggestion, we revised it in **lines 199-200.**

- l. 217: It is strange to call this a meaningful finding given that more or less the same value has been mentioned as result of reference 9 (see also my comment above). The authors should be very clear in distinguishing new and old findings on these data in order to avoid self-plagiarism.

**Response**: Thanks very much for your suggestion.

According to your suggestion, we revised it in **lines 224, and 71-76.**

- It appears dubious that the results and discussion on selective TOPs have been deleted. I understand that selective TOPs are a sensitive topic in China, but the findings cannot be seriously interpreted without explaning this issue properly. In particular, the paragraph in line 217-229 speculates about potential reasons why the PMR of CHDs is so much higher in Hunan (and Zheijang) than in Europe, but these rates just seem to match the rates of selective TOPs. It would e.g. be relevant to mention the PMR for CHDs without selective TOPs and to discuss whether this is comparable to other countries. Further, it should be discussed why there were so much more selective TOPs in Hunan than in Europe, and whether these are more common in rural than urban areas (and if so, why).

**Response**: Thanks very much for your suggestion.

According to your suggestion, we revised it in **lines 157-158, 228-230, and 251-253.**

- I was asking for setting up an online repository which covers both the analysis code and an accompanying data dictionary, see e.g. here: <https://osf.io/qg4u6/>

The respective URL should be mentioned in the Methods section.

**Response**: Thanks very much for your suggestion.

According to your suggestion, we revised it in **lines 324-326.**
